# Environmental Remediation and Conversion of Carbon Dioxide (CO_2_) into Useful Green Products by Accelerated Carbonation Technology

**DOI:** 10.3390/ijerph7010203

**Published:** 2010-01-18

**Authors:** Mihee Lim, Gi-Chun Han, Ji-Whan Ahn, Kwang-Suk You

**Affiliations:** Korea Institute of Geoscience and Mineral Resources (KIGAM), 92 Gwahang-no, Yuseong-gu, Daejeon 305–350, Korea; E-Mails: limmh@paran.com (M.L.); hsue@dreamwiz.com (G.-C.H.); youks@kigam.re.kr (K.-S.Y.)

**Keywords:** carbon dioxide (CO_2_), accelerated carbonation, municipal solid waste, paper mill waste, precipitated calcium carbonate (PCC), recycled paper, thermo-gravimetric (TG) analysis

## Abstract

This paper reviews the application of carbonation technology to the environmental industry as a way of reducing carbon dioxide (CO_2_), a green house gas, including the presentation of related projects of our research group. An alternative technology to very slow natural carbonation is the co-called ‘accelerated carbonation’, which completes its fast reaction within few hours by using pure CO_2_. Carbonation technology is widely applied to solidify or stabilize solid combustion residues from municipal solid wastes, paper mill wastes, *etc*. and contaminated soils, and to manufacture precipitated calcium carbonate (PCC). Carbonated products can be utilized as aggregates in the concrete industry and as alkaline fillers in the paper (or recycled paper) making industry. The quantity of captured CO_2_ in carbonated products can be evaluated by measuring mass loss of heated samples by thermo-gravimetric (TG) analysis. The industrial carbonation technology could contribute to both reduction of CO_2_ emissions and environmental remediation.

## 1. Introduction

The emissions of greenhouse gases such as carbon dioxide (CO_2_), methane (CH_4_), nitrous oxide (N_2_O) and chlorofluorocarbons (CFCs) have increased along with rapid industrialization, so that an increase in the average surface temperature of the Earth over time has resulted. Rising temperatures may, in turn, generate changes in precipitation patterns, storm severity, and sea level, commonly referred to as ‘climate change’ [1].

**Figure 1.**
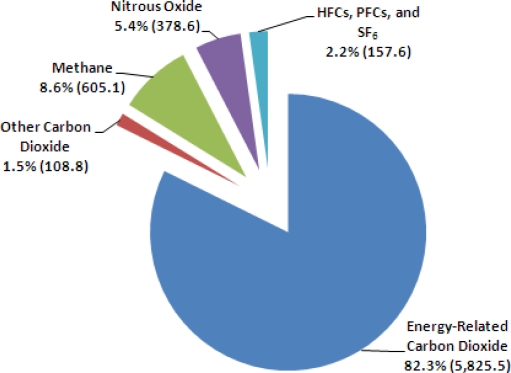
U.S. anthropogenic greenhouse gas emissions by gas in 2006 (million metric tons of carbon dioxide equivalent) (graph modified from [2]).

**Figure 2.**
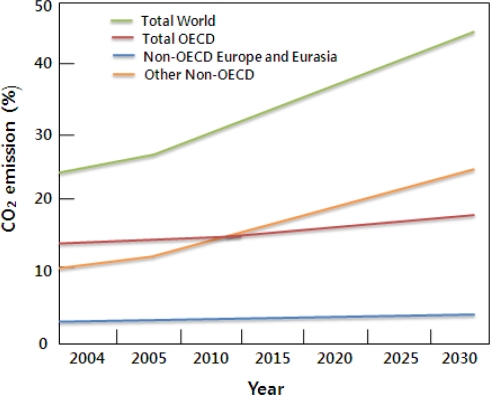
World carbon dioxide emissions by region, 2003–2030 (billion metric tons of carbon dioxide) (graph modified from [3]).

The U.S. Energy Information Administration has stated that greenhouse gases emissions primarily come from the combustion of fossil fuels in energy use. The energy use is largely accompanied by economic growth with short-term fluctuations in its growth rate created by weather patterns affecting heating and cooling needs, as well as changes in the fuel used in electricity generation. As shown in Figure 1, energy-related CO_2_ emissions, resulting from the combustion of petroleum, coal, and natural gas, represented 82% of total U.S. anthropogenic greenhouse gas emissions in 2006 [2]. An annual increase rate in world CO_2_ emissions between 2004 and 2030 is expected to be 1.8% (Figure 2). Much of the increase in these emissions is considered to occur in the developing world such as China and India by fuel economic development with fossil energy. The emissions from the countries outside the Organization for Economic Cooperation and Development (OECD) are expected to grow above the world average at annual 2.6% between 2004 and 2030 [3].

For the Kyoto Protocol in 1997, 163 countries around the world, including South Korea, have agreed to reduce CO_2_ emissions and have been making an effort to develop technologies for its capture, sequestration and utilization, as well as emission reduction [4]. According to the international tendency, the Korean government has recognized the need of more national support for environmental technologies and recently announced an industrial development strategy including research on CO_2_ reduction and utilization under the slogan ‘Low Carbon, Green Growth’ [5]. Therefore, more research in South Korea would be focused on the development of environmental technologies such as CO_2_ fixation and utilization.

As for CO_2_ fixation and utilization, there are physical methods such as capture and sequestration under the ocean, chemical methods such as catalysis/electrochemical reaction, inorganic chemical reaction and carbonation, and biological methods such as forestation/reforestation, plant/algae or microbe usage. Among the prospective technologies for CO_2_ fixation, this paper will be focused on carbonation, which can be a method for both CO_2_ fixation and pollutant stabilization. Carbonation can occur naturally by the reaction between alkaline materials and atmospheric CO_2_, but the natural reaction is very slow [6,7]. Thus, as an alternative technology to the natural carbonation, accelerated carbonation which can complete its reaction within hours has been proposed. The technology has been applied to stabilization of solid residue streams generated from coal fired power stations and other types of combustion residues including de-inking ash, paper mill ash and municipal solid waste ash [8,9]. As well as the stabilization of the wastes by the carbonation, a stabilization technology for heavy metals in contaminated soil by the carbonation using CO_2_ has been also proposed and this technology has revealed as successful in pilot-scale field trials [10,11].

In this paper, we introduce the general mechanism and process of carbonation, and review the application of the technology to environmental remediation and the manufacture of useful products such as carbon captured aggregate and precipitated calcium carbonate (PCC). In addition to the review on previous studies, we also performed a quantitative evaluation of captured CO_2_ in carbonated aggregates. Lastly, the feasibility of the application of carbon captured products in real industry was suggested.

## 2. Carbonation Mechanism and Process

### 2.1. Carbonation Mechanism

**Figure 3.**
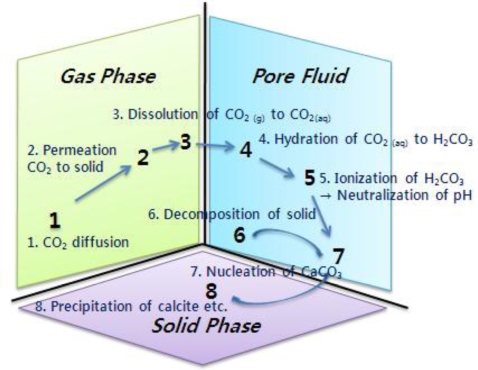
Schematic of accelerated carbonation in eight steps (modified from Marie [12]).

**Figure 4.**
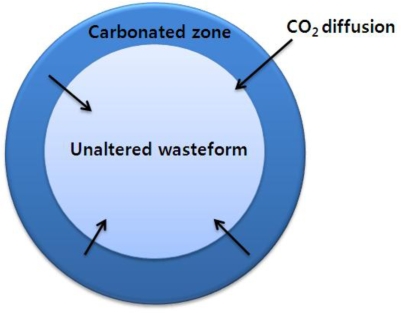
Schematic of carbonation showing a diffusion-controlling reaction (modified from Bin-Shafique [13]).

Carbonation is a strongly exothermic reaction and calcium carbonate (CaCO_3_) is formed by the reaction between cementitious materials and CO_2_ through eight steps, as shown in Figure 3 [11,12]. In general, carbonation is a diffusion-controlling reaction in which the outside surface of the solid is firstly carbonated by the diffusion of CO_2_ into the inner region of the solid and the carbonated area then becomes larger and larger, as shown in Figure 4 [13].

### 2.2. General Carbonation Processes

#### 2.2.1. Natural carbonation

Natural carbonation occurs by the reaction between atmospheric CO_2_ and alkaline materials, which is called ‘weathering’. It is well known that the ‘weathering’ depends on the initial chemical composition, the characteristic of minerals in alkaline materials and the amount of CO_2_ uptake. Natural carbonation generally progresses very slowly over a long term [8].

#### 2.2.2. Accelerated carbonation

The new technology of carbonation is widely used in many industries as an alternative method to natural carbonation which proceeds very slowly in Nature. In accelerated carbonation, CO_2_ in a high purity is artificially injected into solid wastes to make the reaction much faster than that by atmospheric CO_2_, and the reaction can be finalized within a few minutes or hours. This technology is applied to the treatment of solid wastes in which toxic metals are stabilized by carbonated materials, so that the treated solid wastes can be reutilized in other fields [8,11].

### 2.3. Mineral Carbonation Applying the Accelerated Carbonation

Mineral carbonation is one of technologies utilizing CO_2_, and is used to form carbonated materials by the reaction between CO_2_ and Ca or Mg-bound compounds such as wollastonite (CaSiO_3_), olivine (Mg_2_SiO_4_), and serpentine (Mg_3_Si_2_O_5_(OH)_4_) [14,15]. This technology can be also considered as an accelerated carbonation in terms of making the reaction shorter using high purity CO_2_. One of advantages of this technology compared to other CO_2_ storage technologies is that CO_2_ is stably stored in final products such as CaCO_3_ and MgCO_3_. However, this technology needs a process to pressurize Ca or Mg silicates which do not actively react with CO_2_ under normal temperature and pressure and also needs mining development and fragmenting processes. In addition, the carbonation depends on the textural characteristics such as grain size, specific surface area and porosity of Ca or Mg in the sample under normal temperature and pressure. Most of the carbonation rates are under about 550 kg of CO_2_ which is the highest value among those reported [16]. Therefore, this is currently more of a research level field rather than a real industry application due to its low economical efficiency compared to ground, underground, and marine storage [4]. Equations (1)–(6) below present a direct carbonation route [6]. The equations (1) and (2) are processes in which wollastonite (CaSiO_3_) and olivine (Mg_2_SiO_4_) directly react with CO_2_, and the equations (3)–(6) are to increase reaction efficiency by converting those minerals into CaO, Ca(OH)_2_, MgO, and Mg(OH)_2_. The carbonation of CaO and Ca(OH)_2_ are as fast and are completed within a few minutes, whereas the carbonation of Mg(OH)_2_ under dry conditions is very slow for sequestrating CO_2_.

(1) $${\text{CaSiO}}_{3}(\text{s})+{\text{CO}}_{2}(\text{g})+2{\text{H}}_{2}\text{O}(\text{l})\to {\text{CaCO}}_{3}(\text{s})+{\text{H}}_{4}{\text{SiO}}_{4}(\text{aq})\;\Delta \text{H}=-75\;\text{kJ}/\text{mol}\;{\text{CO}}_{2}$$

(2) $$2{\text{Mg}}_{2}{\text{SiO}}_{4}(\text{s})+{\text{CO}}_{2}(\text{g})+2{\text{H}}_{2}\text{O}(\text{l})\to {\text{Mg}}_{3}{\text{Si}}_{2}{\text{O}}_{5}{\left(\text{OH}\right)}_{4}(\text{s})+{\text{MgCO}}_{3}(\text{s})\;\Delta \text{H}=-157\;\text{kJ}/\text{mol}\;{\text{CO}}_{2}$$

(3) $$\text{Mg}{\left(\text{OH}\right)}_{2}(\text{s})+{\text{CO}}_{2}(\text{g})\to {\text{MgCO}}_{3}(\text{s})+{\text{H}}_{2}\text{O}(\text{l}/\text{g})\;\Delta \text{H}=-81\;\text{kJ}/\text{mol}\;{\text{CO}}_{2}$$

(4) $$\text{MgO}(\text{s})+{\text{CO}}_{2}(\text{g})\to {\text{MgCO}}_{3}(\text{s})\;\Delta \text{H}=-118\;\text{kJ}/\text{mol}\;{\text{CO}}_{2}$$

(5) $$\text{Ca}{\left(\text{OH}\right)}_{2}(\text{s})+{\text{CO}}_{2}(\text{g})\to {\text{CaCO}}_{3}(\text{s})+{\text{H}}_{2}\text{O}(\text{l}/\text{g})\;\Delta \text{H}=-113\;\text{kJ}/\text{mol}\;{\text{CO}}_{2}$$

(6) $$\text{CaO}(\text{s})+{\text{CO}}_{2}(\text{g})\to {\text{CaCO}}_{3}(\text{s})\;\Delta \text{H}=-178\;\text{kJ}/\text{mol}\;{\text{CO}}_{2}$$

## 3. Applications of Accelerated Carbonation in the Environmental Industry and Useful Products

### 3.1. Waste Treatment by Accelerated Carbonation

The accelerated carbonation of solid wastes containing alkaline minerals such as Ca and Mg before their landfill treatment is effective for decreasing the mobility of heavy metals by adjusting pH to below 9.5 at which their solubility is lowest. In general, an acidic condition may have the risk of causing releases of heavy metals for a long period, accompanying the decrease in buffering capacity by the decrease in alkalinity, whereas the carbonation at an acidic range can increase the buffering capacity. For instance, Polettini [17] reported that the buffering capacity of bottom ash from solid waste incineration was increased from 0.46~0.48 meq/g to 0.88 meq/g by carbonation. Also, the release potential of heavy metals from the ash is reduced by the carbonation which results in micro-structural changes in the waste and increases the strength of the ash [18].

#### 3.1.1. Municipal solid waste incineration (MSWI) ash

##### Cases of other researchers

Among several solid wastes containing alkaline minerals, the bottom ash from municipal solid waste incineration (MSWI) is one of the most suitable objects to complete the carbonation with CO_2_, so several people around the world have studied the carbonation treatment of MSWI bottom ash using CO_2_. Meimaa *et al*. [19] studied the leachability of Cu and Mo in the interaction of CO_2_ with MSWI bottom ash according to changes in pH and bottom ash mineralogy. They found that the carbonation at pH 8.3 resulted in a reduction of more than 50% in Cu leaching and of less than 3% in Mo leaching. They also reported that the reduction in Cu leaching is attributed to sorption to the neoformed amorphous Al-minerals [19]. Todorovic *et al*. [20] also investigated the stabilizing effect of moderate carbonation (pH 8.28 ± 0.03) on critical contaminants (Cr, Cu, Mo, Sb, Cl^−^, and SO_4_^2−^) through availability and diffusion leaching protocols. In their study, it was found that moderate carbonation decreased the release of Cr, Cu, Mo, and Sb from compacted bottom ash, whereas it was not effective for demobilizing Cl^−^ and SO_4_^2−^ [20]. As a similar study, Gerven *et al*. [21] revealed that a CO_2_ percentage of 10%, a carbonation temperature of 50 °C in the atmosphere, and ash humidity between 13% and 25% were the best carbonation conditions. On the other hand, Rendek *et al*. [22] studied the biodegradation of organic matter in MSWI bottom ash regarding the interaction between the CO_2_ produced by microbial respiration and bottom ash. In the study, they addressed the fact that the organic matter found in bottom ash can provide a substrate for microbial activity, and also the CO_2_ produced by microbial respiration was directly dissolved in bottom ash pore water to be mineralized in carbonate form [22].

##### A case from our research group (Combustion Ash Multi-Processing Lab in KIGAM, South Korea)

###### 1. Carbonation of heavy metals

**Figure 5.**
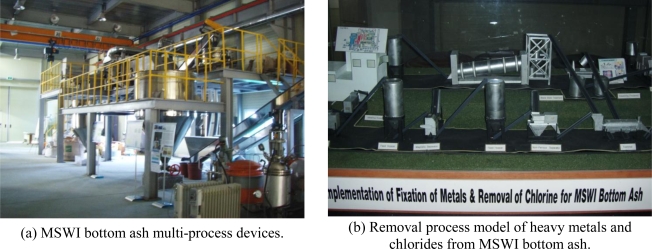
A pilot plant equipped with multi-processes for the recycle of municipal solid waste incineration (MSWI) ash in Korea Institute of Geoscience and Mineral Resources (KIGAM), South Korea (unpublished photos).

In the worldwide trend towards encouraging carbonation treatment of MSWI bottom ash as described above, our research group has also performed carbonation studies using MSWI bottom ash and CO_2_ in order to increase the recycling percentage of the ash and reduce the concentration of atmospheric CO_2_ in Korea [23–25]. In addition, we have established a successful pilot plant for multi-processing of MSWI ash at the Korea Institute of Geoscience and Mineral Resources (KIGAM), Daejeon, South Korea as shown in Figure 5. This pilot plant was the first one in the waste recycling field in Korea and even throughout the Asian countries, and includes multi-processes of particle separation, heavy metal stabilization, chloride removal, and green aggregate/concrete manufacture.

As for our studies on the carbonation, we investigated the carbonation of MSWI bottom ash at different water contents and the stabilization of Cu and Pb in the ash by the carbonation [23]. The result showed that the leaching concentration of Cu and Pb was decreased by the carbonation treatment, as shown in Table 1. In particular, in the case of the carbonation performed at the ratio of 5 (L/S) which is called ‘wet carbonation’, Cu and Pb ions were adsorbed into calcium aluminum compounds, whereas in the case of that at the ratio of 0.2 (L/S), which is called ‘dry carbonation’, particle surfaces are only covered with calcium aluminum compounds of the ash [23].

###### 2. Carbonation of soluble (KCl and NaCl) and insoluble chlorides (Friedel’s salt)

It is reported that South Korean MSWI bottom ash generally contains a high concentration of soluble and insoluble chloride and shows different concentrations, according to its particle size (see Table 2) [26]. Chloride released to the environment in high concentrations has negative effects on the growth of plant and aquatic organisms. Soil microorganism diversity may be decreased by chloride in the soil environment. Soil organic matter (humic substances) can also react with soil inorganic chloride through mediation of microorganisms and exoenzymes. Moreover, mobility of toxic heavy metals from the soil may increase by forming strong complexes with chloride. It is, therefore, necessary to understand environmental impacts by chloride and to develop chloride removal technology [27].

From the study on the removal of chloride from MSWI bottom ash, we found that the chloride in MSWI bottom ash consists of both soluble forms such as KCl and NaCl and insoluble ones such as Friedel’s salt (3CaO·Al_2_O_3_·CaCl_2_·10H_2_O) [24]. Chloride forms a tri-chloro-complex (3CaO·3CaCl_2_·Al_2_O_3_·10H_2_O) by hydration of tri-calcium aluminate (C_3_A) in a solution containing 23% chloride. It also forms a complex consisting of CaCl_2_, Ca(OH)_2_, and CaCO_3_ in a solution of more than 15% calcium chloride (CaCl_2_) at a temperature of less than 20 °C [28]. On the other hand, C_3_A and tetra-calcium aluminoferrite (C_4_AF) form Friedel’s salt (3CaO·Al_2_O_3_·CaCl_2_·10H_2_O) and its ferric form (3CaO·Fe_2_O_3_·CaCl_2_·10H_2_O), respectively, by the reaction with chloride [29,30].

**Figure 6.**
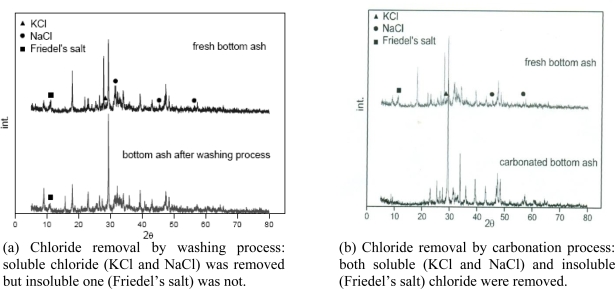
XRD patterns of the bottom ash from municipal solid waste incineration (MSWI) before and after washing (a) and carbonation (b) process (from Jo *et al*. [26] and Ahn *et al*. [24] with permission of *KSGE*).

For the removal of chloride from MSWI bottom ash, we could effectively remove soluble chlorides by a washing process with water, but the process was ineffective for insoluble chloride removal, as shown in Figure 6(a) [26]. However, the accelerated carbonation process was very effective for removing both soluble and insoluble chlorides, as shown in Figure 6(b). Equation (7) expresses the carbonation process of Friedel’s salt [24]:

(7) $$3\text{CaO}\cdot {\text{Al}}_{2}{\text{O}}_{3}\cdot {\text{CaCl}}_{2}\cdot 10{\text{H}}_{2}\text{O}(\text{s})+3{\text{CO}}_{2}(\text{g})\to 3{\text{CaCO}}_{3}(\text{s})+{\text{Al}}_{2}{\text{O}}_{3}(\text{s})+{\text{CaCl}}_{2}(\text{s})+10{\text{H}}_{2}\text{O}(\text{l})$$

###### 3. Conversion of MSWI bottom ash to cement-based material

In addition to the above studies, we also performed an experiment on recycling the ash as a cement-based material by adding a binder [25]. The bottom ash was either washed by water or reacted with CO_2_ bubbles to remove soluble chlorides. After those treatments, the bottom ash was mixed with water and a binder (Portland cement). Then, the mass content of the binder was 10−30% for the ash and the ratio between water and the binder was 0.485.

As shown in Table 3, compressive strengths of the mixtures (mortars) using washed or carbonated bottom ash were higher than those using untreated bottom ash, so that removing chloride in the ash was very effective for its recycling as cement-based material. Based on our formal researches, we are still performing a national research project on the carbonation of MSWI bottom ash and expect to present further novel results soon.

#### 3.1.2. Alkaline paper mill waste

**Figure 7.**
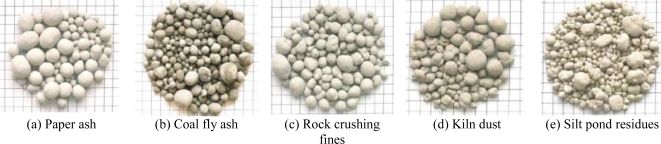
Aggregates manufactured from various industrial wastes by the carbonation process (from Carbon 8 Systems Ltd [30] with permission of P. Carey).

Besides MSWI bottom ash, a study on carbonation using alkaline paper mill waste in high Ca content was performed by Perez-Lopez *et al*. [31]. They investigated the aqueous carbonation mechanisms of an alkaline paper mill waste containing about 55 wt% portlandite [Ca(OH)_2_], as a possible mineralogical CO_2_ sequestration process. They revealed that according to the experimental protocol, the system would capture approx. 218 kg of CO_2_ into stable calcite per one ton of paper waste, independently from initial CO_2_ pressure, and the final product from the carbonation process is a calcite (ca. 100 wt%)-water dispersion [31]. Figure 7(a)−(e) shows aggregates manufactured from various industrial wastes including paper mill ash by the carbonation process [32].

### 3.2. Wastewater Treatment by Accelerated Carbonation

The formation of solid carbonates between aqueous solutions containing divalent cations and CO_2_ is a complicated and important process in Nature and industrial areas. Compared to the aforementioned studies on the carbonation of MSWI bottom ash, however, aqueous carbonation using wastewater is still poorly known and rarely reported in the literature. In this section, we review two representative studies which have reported on the carbonation of cations or anions in wastewater using CO_2_.

As an early study, Enick *et al*. [33] performed the treatment of metal-bearing aqueous waste streams by direct carbonation. In their study, excess liquid CO_2_ was contacted with a plating bath wastewater stream containing 666 mg/L Al and 40 mg/L Zn for 5 min. As a result the concentrations of Al and Zn decreased by 89% and 90%, respectively (see Table 4) and the metal carbonate precipitate was easily filtered [33]. They also announced that the potential of the combined sequestration of the waste is small, but the capacity of the effective treatment of waste streams could contribute to an industrial interest in the development of direct carbonation technology [33].

**Figure 8.**
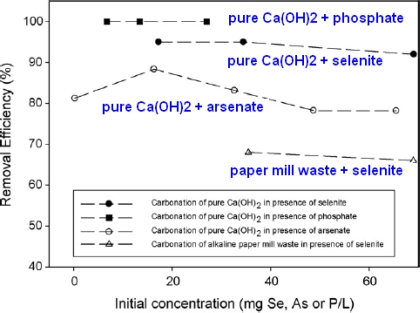
Removal efficiency of selenite, arsenate and phosphate by Ca(OH)_2_ carbonation with compressed CO_2_ (from Montes-Hernandez *et al*. [34] with permission of *Elsevier*).

The carbonation process can be also applied to the removal of toxic oxyanions by incorporation and adsorption into a solid species (e.g., CaCO_3_). It has been reported that the dissolved CO_2_ concentration, pressure and temperature have a great effect on the average particle size, specific surface area, and initial rate of precipitation of the final product [34–36]. Montes-Hernandez *et al*. [34] recently studied the removal of various oxyanions (selenite, selenate, arsenate, phosphate and nitrate) during calcite formation using aqueous carbonation of Ca(OH)_2_ under moderate pressure (P_CO2_≈20 bar) and temperature (30 °C). They also investigated the effects of Ca(OH)_2_ dose (10 and 20 g), Ca(OH)_2_ source (commercial pure material or alkaline paper mill waste), and the initial concentration of oxyanion (from 0 to 70 mg atom/L) for the gas–liquid–solid system. There, they found that the carbonation reaction of Ca(OH)_2_ successfully achieved for eliminating selenite (>90%), arsenate (>78%) and phosphate (≈100%) from synthetic solutions, as shown in Figure 8. However, nitrate and selenate did not show any physical and chemical affinity or effect on the calcite formation [34].

### 3.3. Soil Treatment by Accelerated Carbonation

Heavy metals in contaminated soil can be treated by a traditional stabilization/solidification (S/S) process. In general, Portland cement is added as a binder to improve physical and handling properties of the material and to decrease the mobility of heavy metals. Accelerated carbonation is being currently used as a further process to enhance the traditional S/S technology by achieving the reaction within a few minutes [11,37].

**Figure 9.**
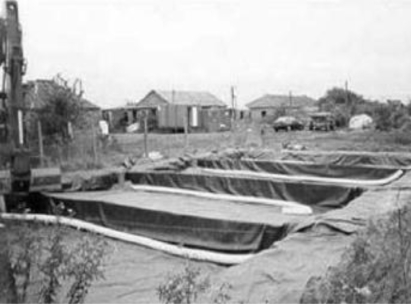
Construction of four pilot-scale cells in the site. Cell 1: untreated soil; Cell 2: EnvirOceM soil; Cell 3: OPC soil; Cell 4: ACT soil (from Jiangying *et al*. [38] with permission of *Elsevier*).

**Figure 10.**
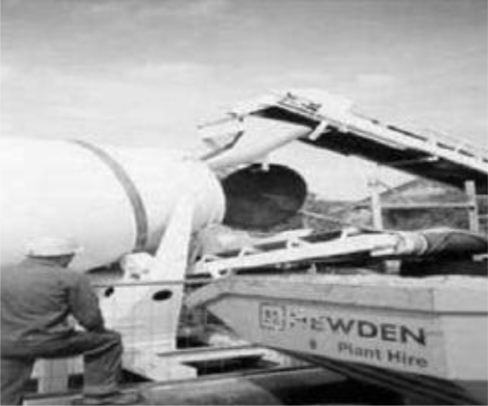
Accelerated carbonation treatment equipment (from Jiangying *et al*. [38] with permission of *Elsevier*).

Accelerated carbonation was successfully applied in pilot-scale field trials in Dartford (Kent, U.K.) in September 2000, as shown in Figures 9 and 10 [11,37]. Heavy metals in the soil on the site were highly remediated with isolating contaminants throughout the site. As a further study performed on the same site, Jiangying and his coworkers [38] investigated on the remediation of contaminated soils in southeast U.K. by comparing traditional S/S and accelerated-carbonated S/S process.

**Figure 11.**
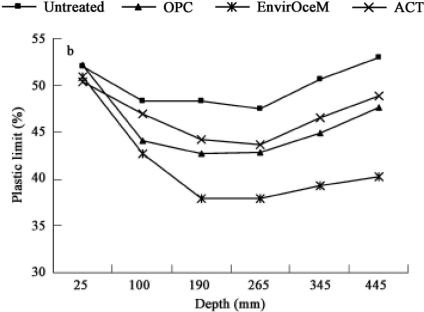
Plastic limit (%) of the soils according to the depth in the four cells: untreated soil > ACT soil > OPC soil > EnvirOceM soil (from Jiangying *et al*. [38] with permission of *Elsevier*).

**Figure 12.**
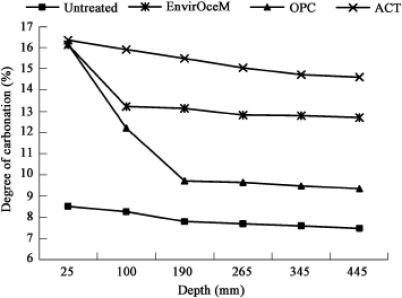
Degrees of carbonation of the soils according to the depth in the four cells: ACT soil > EnvirOceM soil > OPC soil > Untreated soil (from Jiangying *et al*. [38] with permission of *Elsevier*).

Four cell types of soil sample were used: untreated soil, EnvirOceM (proprietary environmental cement) soil, OPC (ordinary Portland cement) soil, and ACT (accelerated carbonation technology) soil. Compared with the traditional cement-based soils, ACT greatly increased the plastic limit (PI (%): potential water content range at which the soil becomes plastic) or density of the treated soil and made PI more stable in the long-term weathering. The increase sequence of PI according to the soil depth is shown in Figure 11: untreated soil > ACT soil > OPC soil > EnvirOceM soil, and also the degree of carbonation in Figure 12: ACT soil > EnvirOceM soil > OPC soil > Untreated soil [38].

**Figure 13.**
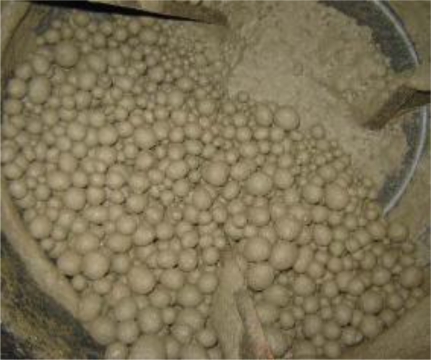
Artificial aggregate in mini concrete mixer (from Melton *et al*. [37] with permission of J.S. Melton).

**Figure 14.**
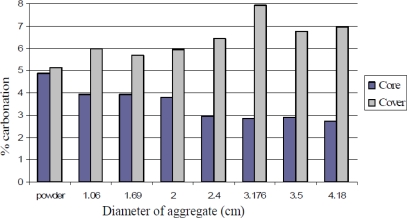
Percentage (%) of carbonated depth in core and cover of the artificial aggregate from the soil (from Melton *et al*. [37] with permission of J.S. Melton).

In addition, a study on producing artificial aggregates from contaminated soil by the accelerated carbonation was performed in USA [37]. In the study, the artificial aggregates were created by mixing soil, water and Portland cement in CO_2_ rich atmosphere, similar to the case using alkaline wastes mentioned in the section “3.1”. Then, around 90–95 % aggregates fall under gravel category (≥4.75 mm) as shown in Figure 13. The study also reported that an increase in diameter of aggregate resulted in a decrease in the diffusion of CO_2_ in the core (see Figure 14) [37].

### 3.4. Synthesis of Precipitated Calcium Carbonate (PCC) by Accelerated Carbonation

#### Cases of other researchers

Carbonation technology utilizing CO_2_ is an industrial platform for transforming CO_2_ emissions into high-value carbonate-based products. This technology has been introduced to produce a high value chemical compound, precipitated calcium carbonate (PCC), currently used in the production of paper, paint, rubber, and plastics [39]. The multi-billion dollar global market for PCC is projected to grow to 10 million tons by 2010 [40]. This growth is mainly attributed to the increase in worldwide paper consumption and construction. Approx. 75% of the produced PCC is expected to be used in the paper industry as a brightness coating and filler [40,41].

**Figure 15.**
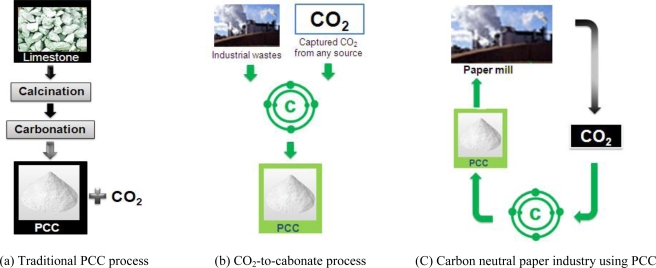
Schematic of PCC manufacture process and application to the paper industry.

Expensive raw materials and a large amount of energy were consumed in the traditional PCC manufacture process, and large amounts of CO_2_ gas were also produced [Figure 15(a)]. On the other hand, a new PCC production process, which is called ‘CO_2_-to-Carbonate technology’, uses inexpensive raw materials such as waste materials from industrial operations and emitted CO_2_, consumes less energy, and decreases CO_2_ by converting it into PCC [Figure 15(b)]. Kakizawa *et al*. [42] suggested an alternative method to CaCO_3_ for CO_2_ sequestration, which is based on extraction of calcium from wollastonite (CaSiO_3_) mineral using acetic acid. Applying this concept to PCC, Teir *et al*. [40] showed that manufacturing PCC through the traditional method will emit CO_2_ as much as 0.21 kg/kg PCC, which assumes oil combustion for lime calcinations, whereas the acetic acid method using wollastonite (CaSiO_3_) will give a net fixation of 0.34 kg CO_2_/kg PCC [41]. A paper mill plant integrated with CO_2_-to-Carbonate process can transform its CO_2_ emissions into PCC used in paper manufacture, so that the process can be one step closer to carbon neutrality [Figure 15(C)].

#### A case from our research group (Eco-PCC Lab in KIGAM, South Korea)

Based on the fact that South Korea has 40 billion tons of the sedimentary rock, our research group was led to develop limestone ore and especially focus on the study of PCC as another research field, along with the aforementioned carbonation of MSWI ash. We have published several scientific articles on PCC crystallization until recently [39,43–51].

**Figure 16.**
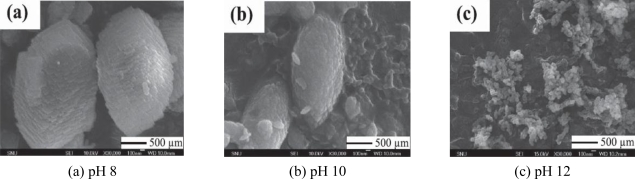
Scanning electron microscope (SEM) images of CaCO_3_ precipitated at various pHs with the addition of NH_4_OH (from Ahn *et al*. [49] with permission of *Resources Processing*).

For instance, we researched the synthesis of CaCO_3_ by carbonation in a pure ethanol and aqueous ethanol solution [46], and investigated the effects on magnesium chloride and organic additives on the synthesis of aragonite PCC [48]. In addition, we studied for the effect of pH and basic additives on the precipitation of CaCO_3_ during the carbonation reaction [49]. In the study, it was found that in the addition of NH_4_OH, one of applied basic additives, the particle size of PCC grew with a decrease in pH and an increase in reaction time [49], as shown in Figure 16. Those studies would contribute to determining optimal conditions of manufacturing commercial PCC in a large scale industry.

**Figure 17.**
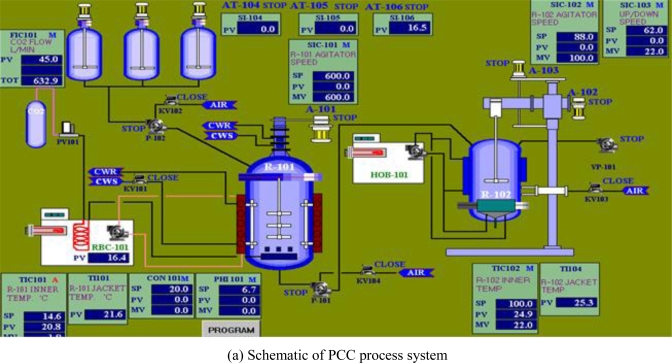

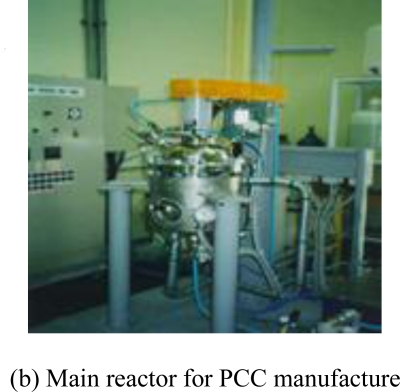
A pilot plant equipped with precipitated calcium carbonate (PCC) process system in Korea Institute of Geoscience and Mineral Resources (KIGAM), South Korea.

Considering the importance of PCC applications to various industries, a PCC pilot plant on a larger scale as shown in Figure 17 was established in KIGAM (South Korea, project by Dr. J.W. Ahn) in 2002. The pilot plant was designed to produce PCC of 99.9% purity and particle sizes in the range of 8−20 μm and 0.3−0.5 μm. The synthesized PCC is currently used in the study of recycled paper making. The technology could be used as leverage, allowing South Korea to participate in developing foreign limestone deposits. Southeast Asian countries such as Vietnam and the Philippines have demonstrated their interest in applying our technology for the development of their own resources [52]. PCC manufacture by the carbonation using CO_2_ could also contribute to the reduction of CO_2_, a green house gas worldwide.

## 4. Quantitative Evaluation of Captured CO_2_ in Carbonated Products

### 

#### 

##### Previous studies on thermo-gravimetric (TG) analysis of CaCO_3_

As mentioned, a variety of research on carbonation has been performed worldwide. It is well known that thermo-gravimetric (TG) analysis is one of the most widely used methods for quantitative evaluation of CO_2_ in carbonated products. The analysis is based on measuring the mass change of a sample caused by heat at temperatures in various ranges. The mass change can be attributed to moisture evaporation or chemical breakdown of compounds into gaseous components [53]. Various temperature ranges (°C) of CaCO_3_ decomposition reported in previous studies are shown in Table 5 [53].

##### TG analysis of carbonated products by our research group (KIGAM, South Korea)

Based on the previous studies of TG analysis, we also performed quantitative evaluation of CO_2_ in carbonated MSWI bottom ash. MSWI bottom ash sample containing water of 30–50% was collected in South Korea and was then dried for a day at 100 °C in an oven to reduce water content to less than 0.1%. Dried sample was sieved into three particle sizes using a standard sieve: (1) below 4 mesh, (2) 4−100 mesh, and (3) over 100 mesh. The bottom ash sample in the particle size of over 100 mesh was selected for the carbonation treatment. Chemical composition (wt. %) of the sample analyzed by X-ray fluorescence (XRF; Supermini(Benchtop), RIGAKU) is shown in Table 6.

**Figure 18.**
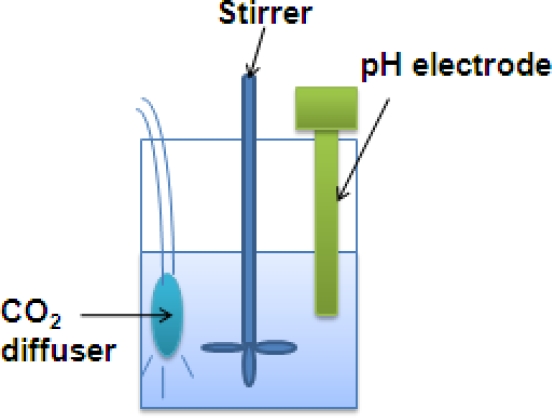
Apparatus of accelerated carbonation at a wet condition (modified from Todorovic *et al*. [20]).

For the carbonation treatment, the sample was suspended with a stirrer in deionized water at a ratio of 1:10 (solid: liquid). Using a gas diffuser, the stirred suspension was treated with pure CO_2_ gas at a rate of 0.5 L/min until pH of the suspension becomes less than 7, at which point the carbonation is completed (Figure 18). After finishing the reaction, the carbonated sample was dried and the mass change of the final product was then measured at various temperatures using a thermo-gravimetric (TG)/derivative TG (DTG) analyzer (TG 209 F3, NETZSCH). The specific analysis conditions were as follows: (1) temperature range: ambient to 1,000 °C, (2) heating and cooling rates: 0.001 and 50 K/min, and (3) cooling time: 20−25 min (from 1,000 to 100 °C).

**Figure 19.**
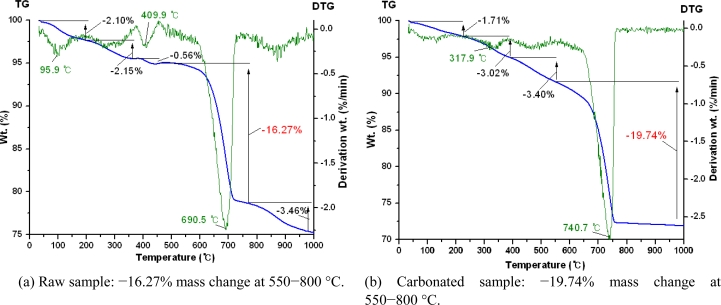
Quantitative evaluation of CO_2_ in municipal solid waste incineration (MSWI) bottom ash before (a) and after accelerated carbonation (b), using TG/DTG analysis at various temperatures (TG = thermo-gravimetric; DTG = derivative thermo-gravimetric) (Unpublished graphs).

As aforementioned, the temperature-mass change curves shown in TG analysis indicate thermal and chemical composition changes of a sample. They also show the thermal properties of intermediate processes that occur during sample heating. The DTG curve from the derivation of a TG curve presents both mass change and decomposition rates in its slope. CaCO_3_ decomposition rate by TG analysis can be considered to determine mass (wt. %) of captured CO_2_ in a sample, because a final product of the carbonation reaction using CO_2_ is CaCO_3_. As other researchers have already reported [56,57], we also assumed that mass change of the TG curve at 550−800 °C can be attributed to CO_2_ release by CaCO_3_ decomposition. As shown in Figure 19, TG curves of raw and carbonated sample present mass losses of −16.27% and −19.74%, respectively, at 550−800 °C. Thus, we confirmed in our experiment that 3.47 wt.% of CO_2_ was captured in the sample by the carbonation treatment, and concluded that the carbonation could contribute to CO_2_ reduction from the atmosphere by sequestrating CO_2_ into stable CaCO_3_.

## 5. Advanced Applications of Carbon Captured Products

### 5.1. Application to the Restoration of Shoreline Environments

#### 

##### A case from Japan (by JFE Steel Corporation)

**Figure 20.**
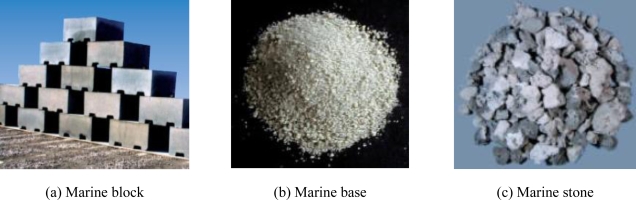
Carbon captured products made from steel slag (JFE Steel [64]).

JFE Steel Corporation in Japan has successfully developed new environmentally-friendly materials made from steel slag. They manufactured three materials from steelmaking slag: an artificial reef for seaweed/coral breeding (Marine Block), a sand-capping material (Marine Base), and a submerged embankment (Marine Stone), as shown in Figure 20 [64].

**Figure 21.**
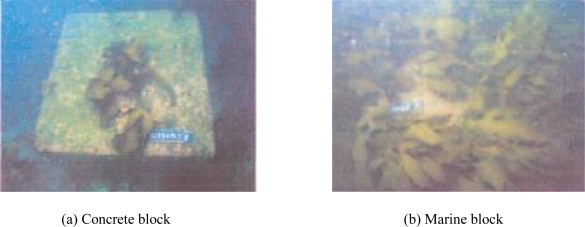
Comparison of seaweed adhesion to normal concrete and marine concrete: more seaweeds were adherent to marine block than concrete block (JFE Steel [64]).

Artificial reefs for seaweed/coral breeding (Marine Block) were manufactured by the carbonation of steelmaking slag using CO_2_. Artificial reefs show a high stability in seawater due to the fact that it consists of CaCO_3_, like shells and coral, and they act as great breeding habitats for seaweeds and coral. Figure 21 shows that more seaweed adhered and grew on the marine block than on normal concrete blocks. A sand-capping material (Marine Base) is produced from granulated blast furnace slag, and consists of calcium oxide and silicon oxide. The elution of orthophosphates and nitrogen compounds, which results in eutrophication, can be prevented by covering the sea bottom with organic materials. Hydrogen sulfide which causes blue tide can also be prevented by the cover. The material size is suitable for bottom-dwelling organism habitats. The submerged embankment (Marine Stone) is also made from steelmaking slag. Marine Stone habitats are more effective than natural stone, because steelmaking slag provides minor elements which are necessary for life [64].

### 5.2. Application of PCC to the Recycled Paper Industry

#### 

##### A case from the United States (by the US Department of Energy)

**Figure 22.**
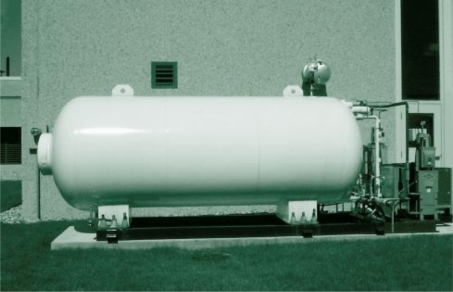
PCC fiber loading system (Forest products, NICE^3^ project [65]).

The United States (U.S.) has been leading a national project on applying PCC to paper recycling. The US Department of Energy has developed a new fiber loading system (developed by Voith Sulzer, Inc.) to improve paper recycling efficiency (see Figure 22). The most effective method for producing PCC as filler is to make it in a satellite plant combined with a paper mill. This also leads to cost savings of 33% compared to producing PCC separately from the paper mill. Fiber loading produces PCC filler in the pulp recycling process at lower cost. For instance, it was reported that producing approx. 500 thousands ton of the recycled paper through the fiber loading system contributes to saving energy of approx. 2.32 × 10^11^ Btu/year (1Btu–0.252kcal) and a waste reduction of approx. 2.32 × 10^11^ Btu/year and CO_2_ emission of approx. 66,805 × 10^3^ ton/year. These favorable effects can create annual cost savings of $3,100,000 [65].

##### A case from South Korea (Eco-PCC Lab, KIGAM)

The Energy Technology Innovation (ETI) R&D Program in the Ministry of Knowledge Economy, South Korea includes a project titled “*Technical Development of Calcium Carbonate Filler used in Eco-friendly Paper*”, which is being performed by our research group at the Eco-PCC Lab in KIGAM. The main objectives of the project are to develop on-site carbonation process for energy efficiency improvement, to synthesize PCC through *in-situ* processes, and to develop effective treatment technologies for paper making process wastewater. Also, it is expected to establish an energy and cost-efficient recycling paper industry applying successful results from this project. From one ton of recycled paper, for example, approx. 4,200 kw of energy per year can be saved because the *in-situ* process, which is a paper making process combined with PCC synthesis, that needs less electricity for drying paper and de-watering sludge. Compared to the traditional paper making process separated from PCC synthesis (*ex-situ* process), the *in-situ* process can also decrease air and water pollution, so it can be considered as an “environment-friendly process”.

Results of this project showed that introducing PCC in the recycled paper making process was effective in improving paper properties (see Table 7).

**Figure 23.**
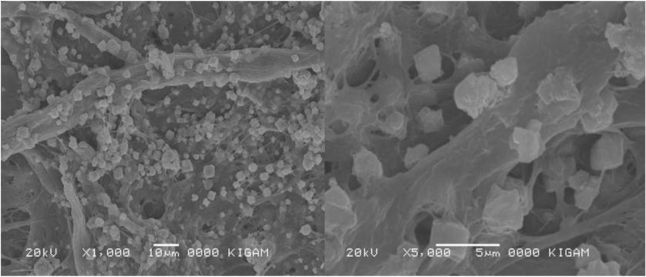
Scanning electronic microscope (SEM) image of microstructure of the recycled paper made from waste pulp (old newspaper) through in-situ process with PCC (10% calcite): PCC filler shows high adhesion to the surface of the pulp (from Ahn *et al*., [66] with permission of *KSGE*).

In particular, the *in-situ* process was more effective for paper properties than the *ex-situ* one. For instance, the whiteness of recycled paper made from waste pulp (old newspaper) without PCC filler was 59.5%, but in the case of using PCC filler the whiteness increased slightly: 61.6% (*ex-situ*) and 64.2% (*in-situ*) [66]. Figure 23 shows scanning electronic microscope (SEM) image of the microstructure of the recycled paper made from waste pulp (old newspaper) through the *in-situ* process with PCC (10% calcite), indicating high adhesion of PCC to the surface of the pulp.

**Figure 24.**
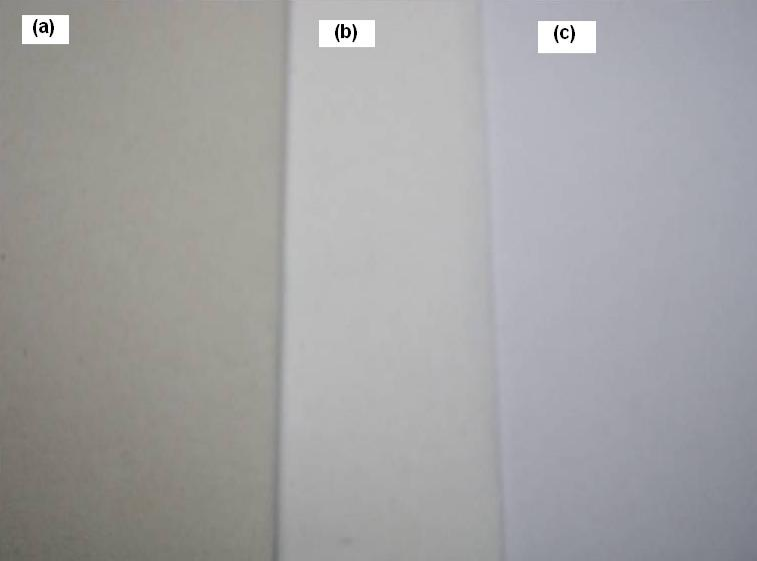
Photos of the recycled papers made from three kinds of waste pulp [(a) old newspaper, (b) white ledger, and (c) printing paper] through in-situ process with PCC (10% calcite): whiteness order (a) < (b) < (c) (unpublished photo).

Figure 24 shows photos of the recycled papers made from three kinds of waste pulp through the *in-situ* process with PCC (10% calcite) in our laboratory, and the order of whiteness degree of the three recycled papers was as follows: (a) the recycled paper made from old newspaper < (b) the recycled paper made from white ledger < (c) the recycled paper made from printing paper.

From the results, we confirmed that optical properties of recycled papers can be more improved by using the *in-situ* process with PCC. In addition, we believe that manufacturing recycled paper using waste pulp and PCC can be a green technology which contributes to reutilizing solid wastes and CO_2_ as a way of environmental conservation.

## 6. Conclusions

Accelerated carbonation is a novel technology to reduce CO_2_ emissions and to convert this substance into various useful green products. The technology is widely being used to solidify or stabilize solid combustion residues and contaminated soils and to manufacture precipitated calcium carbonate (PCC). Carbonated products were also successfully utilized as aggregates in the concrete industry and as alkaline filler in the paper (or recycled paper) making industry. In this review, however, we understood that most studies on the carbonation tend to have focused on the solidification/stabilization of solid combustion residues, and applying the technology to other fields such as wastewater and soil treatment has been less studied until recently. Therefore, we suggest that the technology should be applied to remediate various environmental fields other than those mentioned above and specific reaction mechanisms applied in each field should be also proved. Finally, we expect that this extensive review on the carbonation technology could be useful for other researchers who are interested in it and are making efforts on achieving advanced results.

## Figures and Tables

**Table 1.** Comparison of leaching concentrations (mg/L) of heavy metals and pH values between raw and carbonated bottom ash at wet (L/S = 5) and dry (L/S = 0.2) condition (modified from Ahn *et al*. [23] with permission of *KSGE*).

**Parameter**		**Raw bottom ash**	**Carbonated bottom ash**	

			**Wet carbonation**	**Dry carbonation**
Heavy metal (mg/L)	Cu	1.89	0.05	0.01
	Pb	1.54	<0.01	<0.01
pH		11.74	7.96	9.02

**Table 2.** Chloride concentration (mg/kg) of municipal solid waste incineration (MSWI) bottom ash produced in South Korea according to particle size (mm) (modified from Jo *et al*. [26]).

Particle size	mm	+4.75	2.36–4.75	1.18–2.36	0.6–1.18	0.3–0.6	0.15–0.3	−**0.15**
Chloride	mg/kg	22,499	4,088	7,392	10,412	16,706	25,458	**37,859**

**Table 3.** Compressive strength of mortars (mixtures of the ash, Portland cement, and water) depending on pretreatments of the bottom ash from municipal solid waste incineration (MSWI) (modified from Ahn *et al*. [25] with permission of *KSGE*).

**Sample**	**Pretreatment**	**Compressive strength (kgf/cm^2^)**	
		**3 days**	**7 days**
MSWI bottom ash A	Untreated	170.3	221.3
	Washing	193.8	241.8
	Carbonation	184.8	238.7

MSWI bottom ash B	Untreated	119.5	146.5
	Washing	158.2	184.7
	Carbonation	152.0	173.4

MSWI bottom ash C	Untreated	119.6	160.2
	Washing	153.2	196.8
	Carbonation	149.5	182.4

**Table 4.** Removal efficiency of metals from aqueous waste streams by the carbonation treatment (modified from Enick *et al*. [33]).

	**Unit**	**Metals**				
		**Al**	**Cr**	**Fe**	**Pb**	**Zn**
Feed wastewater	mg/L	666	0.43	5.29	0.28	40
Treated wastewater	mg/L	38	0.20	0.83	0.10	4.27

Removal efficiency	%	94	53	84	64	89

**Table 5.** Temperature range (°C) of Ca(OH)_2_ and CaCO_3_ decomposition reported in previous studies (modified from Haselbach [53] with permission of the *American Society of Civil Engineers*).

	Ca(OH)_2_ TGA decomposition	**CaCO_3_ TGA decomposition**	Aragonite-calcite conversion	Vaterite-calcite conversion
Cole and Kroone (1960) [54]		600–750 poorly crystallized		
		820 well crystallized		
Ramachandran *et al*. (1964) [58]	464 (DTA)	850–950 calcite		
Taylor *et al*. (1985a,b) [59,60]	450–650			
Papadakis *et al*. (1991) [55]	400–500	600–800		
Papadakis *et al*. (1992) [61]	460			
Stern (2001) [62]		827–927 calcite	~460	~350–400
Huijgen *et al*. (2005)-slag [56]		>500		
Huntzinger (2006) [57]	300–500	500–800		
Chang and Chen (2006) [63]	425–550	550–950		

TGA = Thermo-gravimetric analysis; DTA = Derivative thermo-gravimetric analysis.

**Table 6.** Chemical components (wt. %) of the MSWI bottom ash sample analyzed by X-ray fluorescence (XRF) (unpublished data).

**Element**	**Wt.%**	**Element**	**Wt.%**
Na	2.08	Ti	2.11
Mg	2.02	Cr	0.21
Al	4.91	Mn	0.45
Si	7.75	Fe	5.22
P	1.56	Cu	0.65
S	2.38	Zn	1.29
Cl	4.55	Sr	0.22
K	1.89	Ba	1.42
Ca	61.0	Pb	0.28

**Table 7.** Optical properties of the recycled paper made from waste pulp (old newspaper) according to PCC addition and process method (*Ex-situ*: paper making process separated from PCC synthesis, *In-situ*: paper making process combined with PCC synthesis) (modified from Ahn *et al*. [66]).

Optical property	No PCC addition	PCC addition						
		*Ex-situ*						*In-situ*

		Calcite (wt %)			Aragonite (wt %)			Calcite (wt %)
		10	20	30	10	20	30	10
Whiteness (%)	**59.5**	**61.6**	61.6	61	60.4	60.2	61	**64.2**
Opacity (%)	98.6	98.7	98.7	99	98.8	99	99	99.2
